# Evaluating selected gene therapies for cardiovascular diseases: an updated scoping review and evidence map of randomised controlled trials

**DOI:** 10.1136/openhrt-2026-004374

**Published:** 2026-10-20

**Authors:** Eric A Apaydin, Jessica Richard, Skye A Miner, Molly Waymouth, Derek Roberts, Jerry Vockley, Andrea S Richardson

**Affiliations:** 1 RAND Health, RAND Corporation, Santa Monica, California, USA; 2 Department of Medicine, David Geffen School of Medicine, University of California, Los Angeles, Los Angeles, California, USA; 3 Center for Healthcare Innovation, Implementation, and Policy, VA Greater Los Angeles Heatlhcare System, Los Angeles, California, USA; 4 RAND Health, RAND Corporation, Arlington, Virginia, USA; 5 RAND Health, RAND Corporation, Boston, Massachusetts, USA; 6 Department of Pediatrics, School of Medicine, University of Pittsburgh, Pittsburgh, Pennsylvania, USA; 7 RAND Health, RAND Corporation, Pittsburgh, Pennsylvania, USA

**Keywords:** GENETICS, Systematic Reviews as Topic, Quality of Health Care

## Abstract

**Objective:**

Gene therapies are increasingly being developed and evaluated to treat complex diseases with multiple genetic causes, including cardiovascular diseases (CVDs). However, these studies have not yet been reviewed systematically. We aimed to conduct a scoping review of randomised controlled trials of gene therapies for CVDs and create an evidence map of the included literature.

**Methods:**

We searched six databases for studies published from November 2018 to March 2025. Our search yielded 9106 records, and 1864 publications were reviewed at the full-text level.

**Results:**

We included 28 randomised controlled trials (RCTs) evaluating interventions delivered by RNA interference (13 RCTs; 46%), antisense oligonucleotides (9 RCTs; 32%), plasmids (4 RCTs; 14%) and other vectors (adeno-associated virus and adenovirus; 2 RCTs; 7%). Cardiovascular indications included dyslipidaemia (12 RCTs; 43%), ischaemia (4 RCTs; 14%), transthyretin amyloidosis (4 RCTs; 14%), heart failure (2 RCTs; 7%) and other CVDs (6 RCTs; 21%). Samples included an average of 183 participants (range: 5–655); studies followed these participants for an average of 13 months (range: 3–97). Interventions were efficacious in most (22 RCTs; 85%) of the 26 studies that reported efficacy outcomes, but effects lasted less than 1 year in most studies (69%; 18 RCTs). Serious adverse events varied widely by study (range: 0%–62% of intervention group participants), but 19 RCTs reported that less than 10% of intervention group participants experienced a SAE.

**Conclusions:**

Gene therapies for CVDs were reported to be efficacious in most RCTs, but their effects lasted less than 1 year in 69% of studies. Gene therapies for CVDs show promise in their efficacy and relative safety, but more research is needed to evaluate their long-term effects and risks.

**WHAT IS ALREADY KNOWN ON THIS TOPIC**

- Gene therapies are increasingly being evaluated to treat diseases with multiple genetic causes, including cardiovascular diseases (CVDs). However, this evidence base has not yet been reviewed systematically.

**WHAT THIS STUDY ADDS**

- Treatments were efficacious in the majority of the 28 randomised controlled trials (RCTs) we found evaluating gene therapies for CVDs, but these effects lasted less than 1 year in 18 of the 26 of studies (69%) reporting efficacy outcomes. However, these treatments were also relatively safe, as 19 RCTs reported that less than 10% of intervention group participants reported a serious adverse event.

**HOW THIS STUDY MIGHT AFFECT RESEARCH, PRACTICE OR POLICY**

- Gene therapies for CVDs show promise as they are mostly safe and effective, but additional long-term research is needed to understand the durability of their effects and the possibility of post-treatment adverse events.

## Introduction

The burden of cardiovascular diseases (CVD) is very high, affecting nearly 80 million people in the USA and Canada, and almost 800 million people globally.^1^ Treatments for CVD involve a variety of pharmaceutical, surgical and preventative strategies,^2^ but many of these approaches are invasive or require long-term lifestyle change. The use of gene therapies for CVD offers the possibility of single- or limited-dose treatments that have long-term efficacy.^3^ Gene therapies have traditionally focused on rare diseases,^4 5^ but there is increasing development of these treatments for CVD.^6^

In 2026, there are currently 40 cellular and gene therapy products approved for treatment by the US Food and Drug Administration (FDA),^7^ but none are indicated for CVD. However, this may change in the near future, as there are increasing Phase 2 and 3 trials for gene therapies for CVD.^8^ New therapies for CVD could also come to market due to changing regulations. The FDA is aiming to reduce the number of clinical trials needed for new drug authorisations,^9^ and is removing the requirement for trials altogether for personalised treatments with a ‘plausible mechanism’.^10^ Due to both of these factors, research on gene therapies for CVDs may translate to newly approved treatments in the near future.

In 2019, the Patient-Centered Outcomes Research Institute (PCORI) funded two reports that reviewed the evidence for existing gene therapies in the USA and genetic interventions pending potential approval in the near future.^4 5^ 10 gene therapies were FDA-approved at the time these reports were published that found considerable uncertainty around the long-term durability of potentially curative therapies. In 2021, we published an article that expanded on the 2019 reports, providing a broad synthesis of evidence from 119 randomised controlled trials (RCTs) supporting gene therapies.^11^ While most gene therapy interventions improved symptoms, our study found that serious adverse events (SAEs) were common, and the average follow-up duration was 15 months. We called for more research to evaluate the long-term curative potential of gene therapies. In 2023 and 2025, PCORI commissioned two additional reports on the current landscape of evidence for gene therapies.^12 13^ Between these two sets of reports, 10 additional gene therapies were approved by the FDA. The second set of reports found stronger evidence of gene therapy efficacy, but many studies still reported significant safety issues.

There have been several recent reviews focused on a single intervention or disease,^14–16^ but none have systematically reviewed the evidence for gene therapies to treat CVD. Here, we present a scoping review and evidence map of RCTs of select gene therapies for CVD. Scoping reviews provide a broad overview of an evidence base, including areas of strength and areas that need additional research.^17^ However, they do not rate the quality of evidence. Evidence maps are a visual representation of an evidence base and are valuable for quickly providing a broad overview of the literature to a wide variety of stakeholders, including healthcare practitioners, policymakers and funding agencies.^18^

In this article, our objective was to provide a scoping review that broadly summarises recently published RCTs of the most common types of gene therapies for CVD, including treatments delivered by adenoviruses, adeno-associated viruses (AAV), antisense oligonucleotides, RNA interference (RNAi), plasmid DNA and chimeric antigen receptor T-cells (CAR-T). We detail the characteristics of RCTs of gene therapies for CVD and used evidence maps to display the relative efficacy of these therapies compared with the adverse events related to the treatments and the length of follow-up within these studies.

## Methods

### Data sources

We conducted this evidence map based on the review report registered on the Open Science Framework (https://osf.io/eszhy/). Our reporting complies with the Preferred Reporting Items for Systematic Reviews and Meta-Analyses (PRISMA) for Scoping Reviews guidelines.^19^

We collected updated evidence about current use of FDA-approved and unapproved gene therapy interventions in patients from November 2018 to March 2025 and searched the literature for clinical trials in the following databases: PubMed, EMBASE and Web of Science. In addition, we searched ClinicalTrials.gov, a database maintained by the *Journal of Gene Medicine*, and Pink Sheet by Citeline. Published reviews were reference mined, and content experts reviewed and confirmed the completeness of the included studies.

### Search strategy

The search strategy was developed by a librarian and is presented in the online supplemental material 1. This search was conducted as part of a larger scoping review on genetic therapies that included non-RCTs and additional disease indications. RCTs with CVD indication were separated from the overall search for this scoping review. The figure 1 PRISMA flow diagram describes the literature flow.

10.1136/openhrt-2026-004374.supp1 Supplementary data

**Figure 1:**
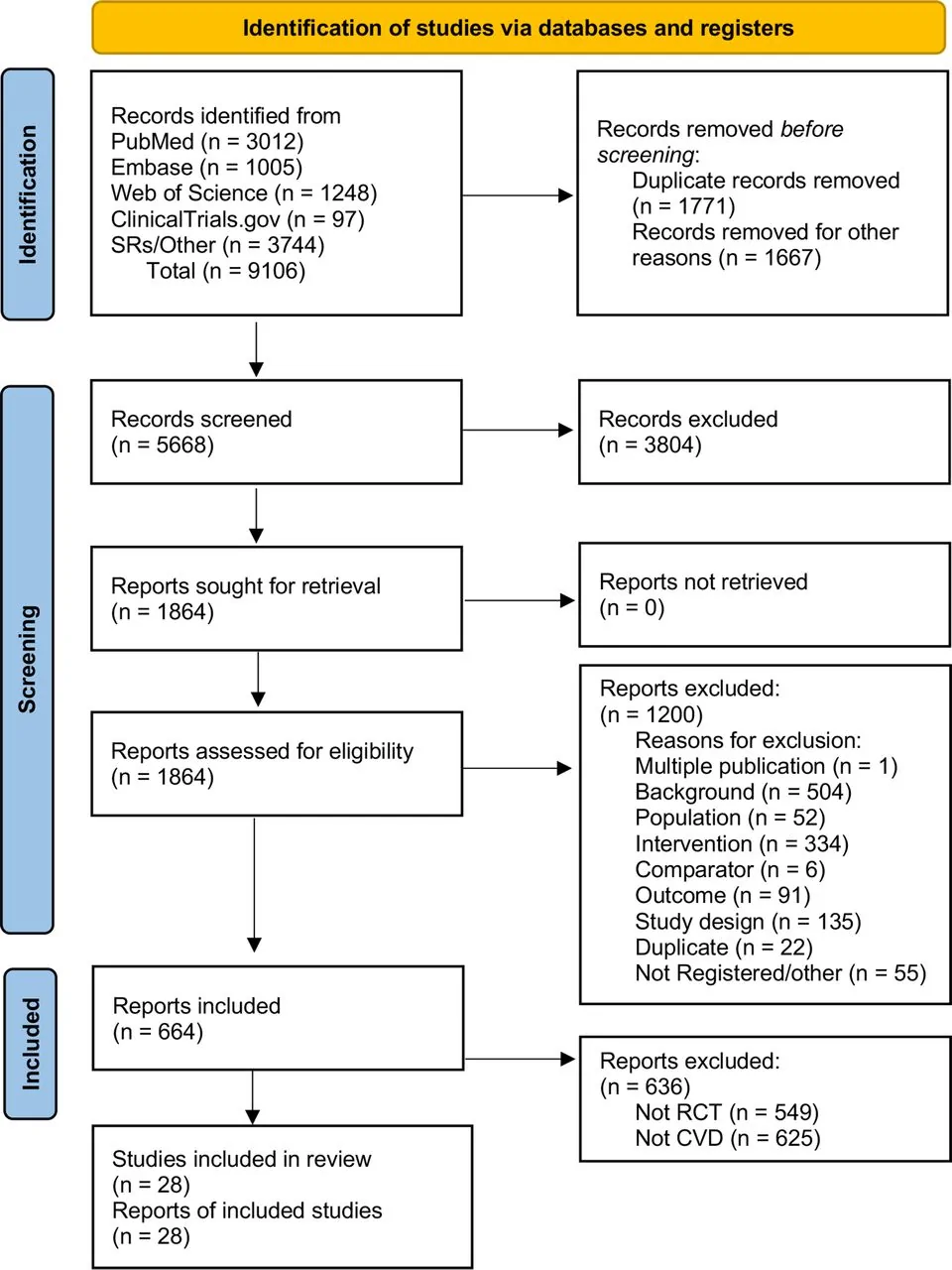
PRISMA flow diagram. Adapted from: Page *et al*.^19^ CVD, cardiovascular diseases; PRISMA, Preferred Reporting Items for Systematic Reviews and Meta-Analyses; RCT, randomised controlled trials; SR, systematic review.

### Screening procedure and eligibility criteria

We obtained any citations as full text if at least one reviewer (EAA, SAM, ASR, MW, DR or JR) deemed them potentially relevant. Eligibility criteria for studies were determined a priori following a Population, Intervention, Comparator, Outcome, Timing, Setting and Study Design framework:

- Population: studies of human participants of all ages who were receiving genetic therapy for a CVD indication were included.
- Interventions: included therapies were plasmid DNA, bacterial vectors, viral vectors including adenovirus, AAV, clustered regularly interspaced short palindromic repeats (CRISPR), CAR-T cell, antisense, RNAi and zinc finger nuclease (ZFN) therapies.
- Comparators: any comparators.
- Outcomes: outcomes included clinical endpoints (eg, mortality, patient-centred and psychosocial outcomes (eg, quality of life), and treatment-associated adverse events/harms (eg, cytokine release syndrome).
- Timing: any study duration and follow-up were eligible.
- Setting: all settings were eligible.
- Study design: RCTs only. Clinical trials without randomisation, single-arm trials and observational studies were excluded. Studies only published in abbreviated format (eg, conference abstracts) were excluded, but RCTs with results in a clinical trial record were included regardless of additional journal publications. Studies published in English between November 2018 and October 2025 were eligible.

### Data abstraction

Data were abstracted using a standardised form. One reviewer (EAA, SAM, ASR, MW, DR or JR) abstracted eligible publications while methodological experts (EAA, SAM or ASR) double-checked these abstractions. Data were categorised based on the previous abstractions from the 2019 reports and their derivative publication.^4 5 11^ Abstracted variables included: CVD indication, intervention type, administration frequency, outcomes measured, sample size, population age range, average age at diagnosis, treatment setting, race/ethnicity, sex, comorbidities and length of follow-up. We also abstracted safety and efficacy according to the following dimensions:

#### Safety

Presence of SAEs related to treatments was captured as reported by individual studies. No SAEs and a lack of SAE assessment were also recorded. Counts of patients with SAEs in intervention and control groups were also abstracted.

#### Efficacy

Efficacy of gene therapy interventions at reducing or eliminating symptoms of the treated disease was classified as follows: durable improvement (reduction in symptoms for at least 1 year with a single course of treatment); sustained improvement (reduction in symptoms for at least 1 year with multiple treatments); improvement (reduction in symptoms for less than 1 year); no improvement (intervention does not improve symptoms compared with control); deterioration (intervention worsens symptoms compared with control, including treatment-related mortality) and unclear. These efficacy categories were initially created and used in our previous evidence map of gene therapies.^11^

### Synthesis

Results were synthesised narratively and displayed in evidence maps. Evidence maps were created using the ggplot2 package (Wickham, Hadley; New York, New York, USA) in R statistical software (R Core Team; Boston, Massachusetts, USA).

## Results

Our search for the scoping review returned 9106 records. After dual title and abstract review, 1864 publications were assessed for eligibility at the full-text level. 28 RCTs with a CVD indication are included in this analysis.^20–47^ This search and selection process is detailed in the literature flow diagram in figure 1, and details of the included RCTs are listed in the evidence table in online supplemental material 2. Safety and efficacy data for the included RCTs are shown as evidence maps in figures 2 and 3.

10.1136/openhrt-2026-004374.supp2 Supplementary data

**Figure 2:**
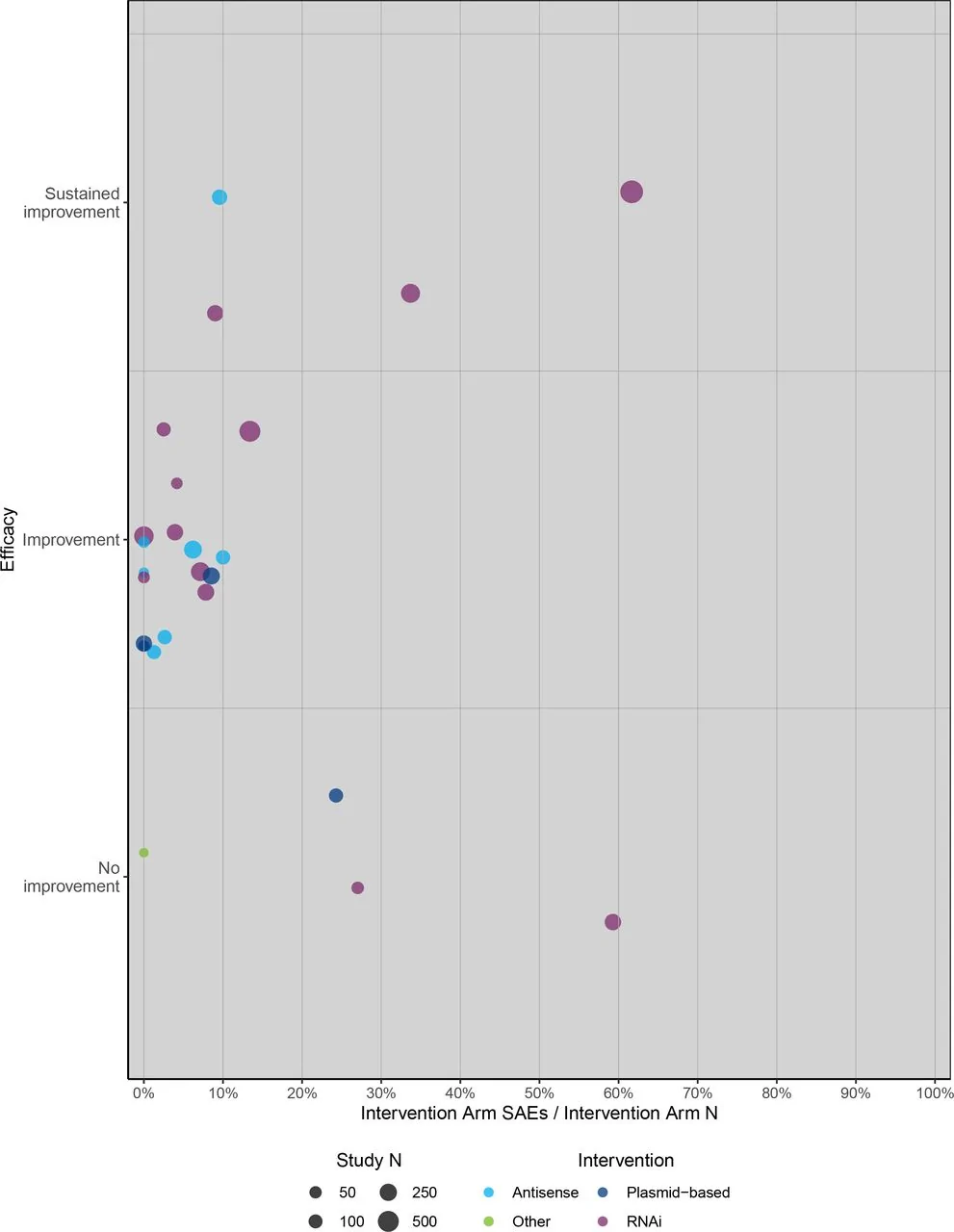
Intervention efficacy and intervention arm SAEs as a percentage of intervention arm sample size. Note: N=25. Map presents studies published between Nov 2018 and Mar 2025. RNAi, RNA interference; SAEs, serious adverse events.

**Figure 3:**
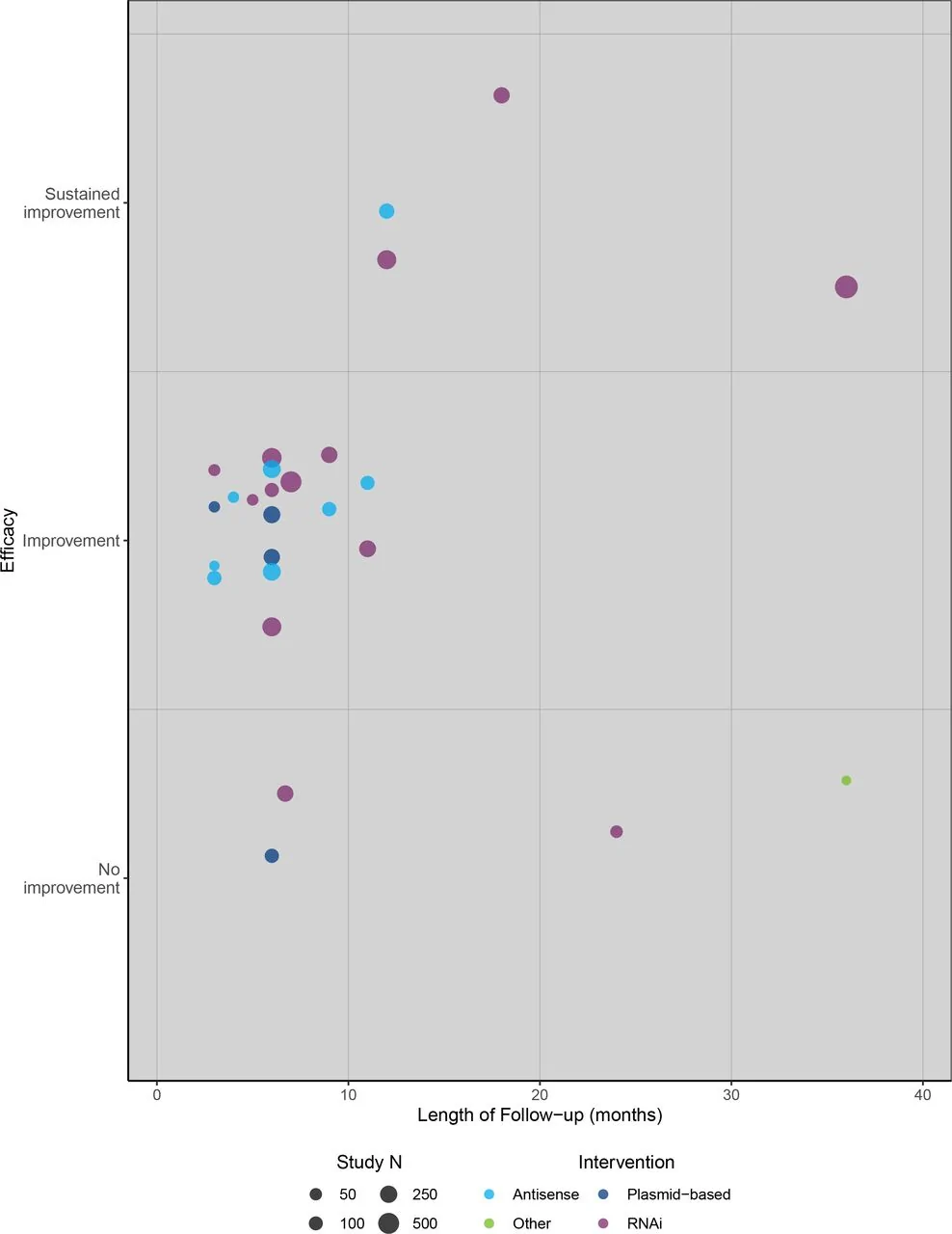
Intervention efficacy and length of follow-up. Map presents studies published between Nov 2018 and Mar 2025. Note: N=26. RNAi, RNA interference.

The included studies examined interventions delivered by RNAi (13 RCTs; 46%)^20 21 30 31 33 35 37–41 43 47^ followed by antisense oligonucleotides (9 RCTs; 32%)^28 29 32 34 36 42 44–46^ and plasmid-based therapies (4 RCTs; 14%).^22–25^ The remaining studies (2 RCTs; 7%)^26 27^ examined interventions delivered by other therapies (AAV and adenovirus). No RCTs assessing bacterial-based, CRISPR-based or ZFN gene therapies met our inclusion criteria for RCTs studying CVD.

Indications included dyslipidaemia (12 RCTs; 43%),^21 28 29 32 35 39 41–44 46 47^ ischaemia (4 RCTs; 14%),^22–25^ transthyretin amyloidosis (ATTR; 4 RCTs; 14%),^31 33 37 40^ heart failure (2 RCTs; 7%)^27 45^ and other CVDs (6 RCTs; 21%)^20 26 30 34 36 38^ such as hypertension, atherosclerotic CVD and other lipid disorders.

Study sample sizes ranged from 5 to 655 participants (mean: 183; SD: 160), 20–47and follow-up time ranged from 3 to 97 months (mean: 13 months; SD: 19 months; figure 3). Treatment administration frequency was most often in limited, multiple doses (21 RCTs; 75%),^20 21 23 24 28 29 31–38 40 42–47^ and other studies administered treatment just once (7 RCTs; 25%).^22 25–27 30 39 41^ All studies focused on adults 18 years and older, and the average proportion of female participants was 0.31 (SD: 0.19; range: 0–0.73). Additional variables that were less frequently reported (specific efficacy outcomes measured, population age range, treatment setting, race/ethnicity and comorbidities) are described in the evidence table (online supplemental file 2).

27^20–45 47^ of the 28 studies (96%) assessed safety outcomes. Most of these studies reported that SAEs occurred in both the intervention and comparator groups (17 RCTs; 63%),^21 23 25 28–31 33 35 37 38 40 43 44 47^ and a few additional studies reported SAE occurrence in the intervention group only (3 RCTs; 11%).^34 36 41^ The remaining studies evaluating SAEs (7 RCTs; 26%)^20 22 24 27 32 39 45^ did not report any occurrences in either group. The proportion of studies reporting SAE occurrence was highest for RNAi therapies (11 RCTs; 85%).^21 30 31 33 35 37 38 40 41 43 47^ SAEs occurred in about half of studies of antisense therapies (5 RCTs; 56%)^28 29 34 36 44^ and half of studies evaluating plasmid-based therapies (2 RCTs; 50%).^23 25^ SAEs occurred in one of the two the studies evaluating other interventions.^26^ Among all studies, reported SAEs were not always specified but included death, cardiac failure and atrial fibrillation.

Genetic therapies were described as efficacious in the majority (22 RCTs; 85%)^20–24 29–36 38–41 43–46^ of the 26 studies that reported statistically tested efficacy outcomes across the intervention categories. Interventions were most reported as improving symptoms for less than 1 year (18 RCTs; 69%),^20–24 28 30 32 34–36 38 39 41 43–46^ followed by sustained improvement of symptoms for more than 1 year with multiple courses of treatment (4 RCTs; 15%).^29 31 33 40^ No improvement was observed in four studies (15%).^25 27 37 47^ Neither durable improvement nor deterioration were observed in any studies.

Our evidence maps plot these categories of efficacy against adverse event occurrence (figure 2) and length of follow-up (figure 3) and further distinguish studies by intervention type and sample size. These figures only include 25 or 26 of the 28 studies in this review, as two studies did not assess efficacy^26 42^ and one study did not assess adverse events in the intervention arm.^46^ Of all studies reporting safety,^20–45 47^ 0%–62% of intervention group participants reported an SAE. 19 studies reported SAEs in less than 10% of intervention group participants.^20–24 27–30 32–36 39 41–43 45^ Most studies (17 RCTs; 68%)^20–24 28 30 32 34–36 38 39 41 43–45^ included in figure 2 reported improvement in symptoms for less than 1 year, and nearly all of these studies reported SAEs for less than 10% of participants in intervention groups (15 RCTs; 88%).^20–24 28 30 32 34–36 39 41 43 45^ Studies of therapies that had sustained or no improvement had much greater variation in SAEs. However, as shown in figure 3, these estimates of improvement may have been constrained by follow-up. Most studies that showed improvement only followed patients for less than 10 months (16 RCTs; 89%).^20–24 28 30 32 36 38 39 41 43–46^ Studies reporting sustained, or no improvement, followed participants for 6–36 months.^25 27 29 31 33 37 40 47^

## Discussion

Our review identified 28 RCTs evaluating gene therapies for CVD. These therapies were efficacious in most studies, but many showed that symptom improvement lasted less than a year or required multiple treatments for longer-lasting effects. These therapies also had significant safety issues, with most studies reporting SAEs in intervention groups. However, the majority of studies reported SAEs in fewer than 10% of participants in the intervention groups. Intervention efficacy was unrelated to SAEs and follow-up length. Long-term SAEs may have been missed in studies with a short follow-up. Most gene therapies for CVDs appear safe and efficacious, but long-term safety and effects remained mostly unmeasured.

Gene therapies for CVDs are still experimental, but the literature we reviewed shows that most therapies were efficacious for less than 1 year. This data on efficacy might have been censored, as the included studies followed patients for only 12 months on average. Follow-up was relatively short in most studies in our review, with an average of 12 months. This is shorter than the average of 15 months of follow-up we found in our previous review of gene therapies,^11^ and still represents a challenge to understanding the long-term safety of these treatments. Gene- or virus-related adverse events, such as unintended gene expression, off-target genetic effects or latent viral vector expression, may occur in patients long after these therapies have produced their intended effects. The FDA has recently called for gene therapy trials with up to 15 years of follow-up,^48^ as many of these evaluations end too early to evaluate the long-term effects of gene therapies. The study with the longest follow-up in our review^26^ observed patients for only half the recommended length, indicating significant room for improvement in the duration of follow-up for these and future gene therapies for CVDs. A recent review of trials in ClinicalTrials.gov found that there is a lack of long-term trials evaluating gene therapies for CVDs.^49^ Only two trial records related to CVDs were found, including a study following patients who received gene therapy for type 2 diabetes for 10 years,^50^ and a study following patients who received a gene therapy for CVD for 15 years.^51^ Additional long-term studies of gene therapies for CVDs need to be started now for there to be long-term efficacy data for these treatments in the near future.

Still, these limited-dose therapies may be preferable for many patients compared with conventional treatments. Our review found gene therapy treatments for heart failure, dyslipidaemia, ischaemia, diabetes, ATTR and other CVD indications. Conventionally, all of these conditions are treated by long-term medication use or lifestyle modification. ATTR is conventionally treated with heart failure therapies (eg, mineralocorticoid receptor antagonists and sodium-glucose cotransporter 2 inhibitors) or transthyretin stabilisers or silencers,^52^ and diabetes is typically treated with insulin, metformin and other glucose-lowering medications, as well as new glucagon-like peptide 1 (GLP-1) receptor agonists.^53^ Heart failure, dyslipidaemia and ischaemia are typically treated with antihypertensive medications, statins and antithrombotic medications.^2^ Lifestyle changes can also help manage all of these conditions, other than ATTR. However, lifestyle changes and long-term medication adherence are difficult to sustain for many patients. The rapid increase in GLP-1 receptor agonists for obesity and type-2 diabetes prescriptions,^54^ especially those that are efficacious with less-than-daily administrations like semaglutides,^55^ indicates a patient interest in efficacious treatments for chronic conditions with a lower patient burden. Gene therapies could offer a less burdensome treatment for CVDs than lifestyle changes and conventional medications.

Our review also showed that most therapies were relatively safe. In 19 studies, less than 10% of participants in the intervention group reported an SAE. Of course, the frequency of these events is higher than that of those that would be experienced by most patients taking conventional medications or undertaking lifestyle changes to treat CVDs. The maximum acceptable risk (MAR), or the highest risk acceptable to patients for some treatment benefit, varies widely for patients and providers by treatment benefit, condition and risk.^56–58^ MAR is typically measured by surveying healthy individuals or patients with a disease about the risk of certain adverse events, including death, that they would be willing to endure to avoid, slow or cure a particular disease.^59^ These proposed adverse events are theoretical, but this approach offers a way for individuals to directly express their preference for the trade-offs between the benefits and risks of a particular treatment. MAR research for gene therapies is scant, but several surveys^56 60^ have shown that patients with Duchenne Muscular Dystrophy (DMD) are willing to undertake a 3.7%–5.7% risk of death from gene therapy to enjoy at least 10 years of improved muscle function. Nonetheless, DMD is a degenerative disease that often ends in early death,^61^ so these patients likely have a much higher MAR for gene therapy-related adverse events than patients with CVDs. There is currently no research on MAR for gene therapies for CVDs, as these gene therapies are still experimental and conventional treatments are relatively low-risk. However, patients may be willing to accept some risk for curative or long-duration gene therapies, although this risk tolerance is likely lower than in patients with more severe diseases.

This review encompassed nearly 7 years of literature from six major databases but remains constrained by several limitations. First, we did not perform a meta-analysis of the effects of gene therapies on CVD symptoms. This was not a feasible approach given the heterogeneity of CVD conditions and associated outcomes we found in the literature, and so it likely will not be possible until additional research on each condition is conducted. Second, we were not able to distinguish between the harms of viral vectors and the effects of gene therapies themselves. The wide variety of vectors and therapies, coupled with the limited number of studies available, makes it impossible to analyse these differences. Third, we analysed all outcomes and lengths of follow-up together, regardless of condition or type of outcome. This likely biases effectiveness estimates towards outcomes that are quickly resolved, given that most RCTs had follow-up periods of less than 12 months. As noted above, more detailed, outcome-specific analyses will only be possible until additional research on the efficacy of gene therapies for each condition is conducted. Finally, we had a relatively broad definition of ‘cardiovascular disease’, grouping disparate conditions like ATTR and hyperlipidaemia. This is not ideal, as these conditions vary widely in their severity, but since the goal of this review was to analyse only RCTs, we had to group the limited available evidence to conduct our analyses. Additional research is needed to accurately estimate the efficacy of gene therapies for each CVD condition.

Our scoping review of RCTs of gene therapies for CVDs showed that most therapies have an effect for less than a year, and that fewer than 10% of participants in intervention groups in most studies reported SAEs. However, these safety and efficacy data were limited by short follow-up, averaging only 13 months. Still, these relatively safe and efficacious gene therapies show promise and could offer better treatments or even cures for CVD in the future. More research is needed to truly understand how these gene therapies affect patients over a longer period of time.

## Data Availability

All data relevant to the study are included in the article or uploaded as supplementary information. All data analysed in this review was drawn from published studies and is available in this article.
