# Supplementary material for: Evaluating selected gene therapies for cardiovascular diseases: an updated scoping review and evidence map of randomised controlled trials
Source: Open Heart. 2026 Oct 20;13(2):e004374. doi: 10.1136/openhrt-2026-004374 (PMC13711567; doi:10.1136/openhrt-2026-004374)
Supplement: Supplementary file 1 [file openhrt-13-2-s001.pdf]

Supplementary Material 1: Search strategies

Section 1. Searches of the PubMed Database

	**PubMed – Humans; English limits applied**	
#1	IMLYGIC[tiab] OR "Talimogene laherparepvec"[tiab] OR T-VEC[tiab] OR LUXTURNA[tiab] OR "Voretigene neparvovec"[tiab] OR Zolgensma[tiab] OR "Onasemnogene abeparvovec"[tiab] OR CRISPR*[tiab] OR "clustered regularly interspaced short palindromic repeats"[tiab] OR "Clustered Regularly Interspaced Short Palindromic Repeats"[Mesh]	44,583
#2	"trial protocol"[ti] OR "study protocol"[ti] OR Clinical Trial Protocol[pt]	25,999
#3	#1 NOT #2	44,580
#4	"observational stud*"[tiab] OR "cohort stud*"[tiab] OR "cohort analys*"[tiab] OR "incidence stud*"[tiab] OR "follow up"[tiab] OR followup[tiab] OR "comparative stud*"[tiab] OR "pre post"[tiab] OR "before after"[tiab] OR "longitudinal stud*"[tiab] OR "longitudinal analys*"[tiab] OR "prospective stud*"[tiab] OR "retrospective stud*"[tiab] OR questionnaire*[tiab] OR survey[tiab] OR "panel stud*"[tiab] OR "Observational Study"[pt] OR "Epidemiologic Studies"[MAJR:noExp] OR "Retrospective Studies"[Mesh] OR "Cohort Studies"[Mesh] OR "Follow-Up Studies"[Mesh] OR "Longitudinal Studies"[Mesh] OR "Prospective Studies"[Mesh] OR "Retrospective Studies"[Mesh] OR "Controlled Before-After Studies"[Mesh]	4,682,171
#5	#3 AND #4	721
#6	rodent*[tiab] OR mouse[tiab] OR mice[tiab] OR animal*[tiab] OR pig[tiab] OR pigs[tiab] OR porcine*[tiab] OR "non human*"[tiab] OR "nonhuman*"[tiab]	2,873,086
#7	#5 NOT #6	591
#8	(2018/11/1:2024/1/20[pdat])	7,752,206
#9	#7 AND #8	484
#10	#9 AND ((humans[Filter]) AND (english[Filter]))	**315**
	Total (Post-deduplication)	**315**

	**PubMed – Humans; English limits applied**	
#1	(gene-editing[tiab] OR "gene editing"[tiab]) AND (therap*[tiab] OR treatment*[tiab])	4,663
#2	"gene therapy"[tiab] OR "gene therapies"[tiab] OR "gene engineering"[tiab] OR "gene transfer*"[tiab]	89,801
#3	"Gene Editing/ethics"[mh] OR "Gene Editing/instrumentation"[mh] OR "Gene Editing/legislation and jurisprudence"[mh] OR "Gene Editing/standards"[mh] OR "Gene Editing/statistics and numerical data"[mh] OR "Gene Editing/trends"[mh] OR "Genetic Therapy/adverse effects"[mh] OR "Genetic Therapy/classification"[mh] OR "Genetic Therapy/epidemiology"[mh] OR "Genetic Therapy/ethics"[mh] OR "Genetic Therapy/instrumentation"[mh] OR "Genetic Therapy/legislation	29,115

	and jurisprudence"[mh] OR "Genetic Therapy/methods"[mh] OR "Genetic Therapy/mortality"[mh] OR "Genetic Therapy/nursing"[mh] OR "Genetic Therapy/psychology"[mh] OR "Genetic Therapy/standards"[mh] OR "Genetic Therapy/statistics and numerical data"[mh] OR "Genetic Therapy/therapeutic use"[mh] OR "Genetic Therapy/therapy"[mh] OR "Genetic Therapy/trends"[mh] OR "Gene Transfer Techniques/adverse effects"[mh] OR "Gene Transfer Techniques/drug effects"[mh] OR "Gene Transfer Techniques/immunology"[mh] OR "Gene Transfer Techniques/therapy"[mh] OR "Gene Transfer, Horizontal/adverse effects"[mh] OR "Gene Transfer, Horizontal/drug effects"[mh] OR "Gene Transfer, Horizontal/immunology"[mh] OR "Gene Transfer, Horizontal/therapeutic use"[mh]	
#4	"betibeglogene autotemcel"[tiab] OR zynteglo[tiab] OR "elivaldogene autotemcel"[tiab] OR skysona[tiab] OR "adeno associated virus"[tiab] OR "adenoassociated virus"[tiab] OR "adeno-associated virus"[tiab] OR CRISPR*[tiab] OR "clustered regularly interspaced short palindromic repeats"[tiab] OR "Clustered Regularly Interspaced Short Palindromic Repeats"[Mesh] OR "betibeglogene autotemcel" [Supplementary Concept]	55,513
#5	#1 OR #2 OR #3 OR #4	148,572
#6	"trial protocol"[ti] OR "study protocol"[ti] OR Clinical Trial Protocol[pt]	25,998
#7	#5 NOT #6	148,556
#8	rodent*[tiab] OR mouse[tiab] OR mice[tiab] OR animal*[tiab] OR pig[tiab] OR pigs[tiab] OR porcine*[tiab] OR "non human*"[tiab] OR "nonhuman*"[tiab]	2,873,039
#9	#7 NOT #8	104,510
#10	"clinical trial"[pt] OR "randomized controlled trial"[pt] OR "controlled clinical trial"[pt] OR "Clinical Study" [pt:NoExp] OR "randomized controlled trials as topic"[mh] OR "Double-Blind Method"[mh] OR "randomized controlled trial*"[tiab] OR (randomized[ti] AND controlled[ti] AND trial[ti]) OR "randomized trial*"[tiab] OR "randomised controlled trial*"[tiab] OR (randomised[ti] AND controlled[ti] AND trial[ti]) OR "randomised trial*"[ti] OR "clinical trial*"[ti] OR "double blind*"[ti] OR "clinical stud*"[ti] OR "placebo controlled trial*"[tiab] OR "systematic review"[pt] OR "Systematic Reviews as Topic"[mh] OR "systematic review*"[ti] OR "review of literature"[ti] OR "rapid review*"[ti] OR "rapid evidence assessment*"[ti] OR "scoping review*"[ti] OR "mapping review*"[ti] OR "literature review*"[ti] OR "Pragmatic Clinical Trial"[pt] OR "Clinical Trial, Phase IV"[pt] OR "Clinical Trial, Phase III"[pt] OR "Clinical Trial, Phase II"[pt] OR "Clinical Trial, Phase I"[pt]	1,721,946
#11	#9 AND #10	1,830
#12	(2018/1/1:2024/11/20[pdat])	632
#12	(#11 AND #12) AND ((humans[Filter]) AND (english[Filter]))	**505**
	Total (Post-deduplication)	**504**

	**PubMed – Humans; English limits applied**	
#1	Lenmeldy[tiab] OR olezarsen[tiab] OR Vupanorsen[tiab] OR IONIS-APOCIII-LRx[tiab] OR AKCEA-APOCIII-LRx[tiab] OR Libmeldy[tiab] OR "atidarsagene autotemcel"[tiab] OR "Beqvez"[tiab] OR "Fidanacogene elaparvovec"[tiab] OR "Fidanacogene elaparvovec-dzkt"[tiab]	48
#2	"trial protocol"[ti] OR "study protocol"[ti] OR Clinical Trial Protocol[pt]	26,660
#3	#1 NOT #2	48
#4	rodent*[tiab] OR mouse[tiab] OR mice[tiab] OR animal*[tiab] OR pig[tiab] OR pigs[tiab] OR porcine*[tiab] OR "non human*"[tiab] OR "nonhuman*"[tiab]	2,892,687
#5	#3 NOT #4	46
#6	"clinical trial"[pt] OR "randomized controlled trial"[pt] OR "controlled clinical trial"[pt] OR "Clinical Study" [pt:NoExp] OR "randomized controlled trials as topic"[mh] OR "Double-Blind Method"[mh] OR "randomized controlled trial*"[tiab] OR (randomized[ti] AND controlled[ti] AND trial[ti]) OR "randomized trial*"[tiab] OR "randomised controlled trial*"[tiab] OR (randomised[ti] AND controlled[ti] AND trial[ti]) OR "randomised trial*"[ti] OR "clinical trial*"[ti] OR "double blind*"[ti] OR "clinical stud*"[ti] OR "placebo controlled trial*"[tiab] OR "systematic review"[pt] OR "Systematic Reviews as Topic"[mh] OR "systematic review*"[ti] OR "review of literature"[ti] OR "rapid review*"[ti] OR "rapid evidence assessment*"[ti] OR "scoping review*"[ti] OR "mapping review*"[ti] OR "literature review*"[ti] OR "Pragmatic Clinical Trial"[pt] OR "Clinical Trial, Phase IV"[pt] OR "Clinical Trial, Phase III"[pt] OR "Clinical Trial, Phase II"[pt] OR "Clinical Trial, Phase I"[pt]	1,739,143
#7	#5 AND #6	14
#8	(2018/1/1:2024/11/20[pdat])	9,160,910
#9	(#7 AND #8) AND ((humans[Filter]) AND (english[Filter]))	**11**

**No.**	**PubMed – Humans; English limits applied**	**Results**
1	(gene-editing[tiab] OR "gene editing"[tiab]) AND (therap*[tiab] OR treatment*[tiab])	5829
2	"gene therapy"[tiab] OR "gene therapies"[tiab] OR "gene engineering"[tiab] OR "gene transfer*"[tiab]	94 586
3	"Gene Editing/ethics"[mh] OR "Gene Editing/instrumentation"[mh] OR "Gene Editing/legislation and jurisprudence"[mh] OR "Gene Editing/standards"[mh] OR "Gene Editing/statistics and numerical data"[mh] OR "Gene Editing/trends"[mh] OR "Genetic Therapy/adverse effects"[mh] OR "Genetic Therapy/classification"[mh] OR "Genetic Therapy/epidemiology"[mh] OR "Genetic Therapy/ethics"[mh] OR "Genetic Therapy/instrumentation"[mh] OR "Genetic Therapy/legislation and	30 617

	jurisprudence"[mh] OR "Genetic Therapy/methods"[mh] OR "Genetic Therapy/mortality"[mh] OR "Genetic Therapy/nursing"[mh] OR "Genetic Therapy/psychology"[mh] OR "Genetic Therapy/standards"[mh] OR "Genetic Therapy/statistics and numerical data"[mh] OR "Genetic Therapy/therapeutic use"[mh] OR "Genetic Therapy/therapy"[mh] OR "Genetic Therapy/trends"[mh] OR "Gene Transfer Techniques/adverse effects"[mh] OR "Gene Transfer Techniques/drug effects"[mh] OR "Gene Transfer Techniques/immunology"[mh] OR "Gene Transfer Techniques/therapy"[mh] OR "Gene Transfer, Horizontal/adverse effects"[mh] OR "Gene Transfer, Horizontal/drug effects"[mh] OR "Gene Transfer, Horizontal/immunology"[mh] OR "Gene Transfer, Horizontal/therapeutic use"[mh]	
4	(human* AND "gene editing technology"[tiab:~2]) OR (human* AND "gene editing technologies"[tiab:~2]) OR "RNA interference"[tiab] OR RNAi[tiab] OR siRNA[tiab] OR "RNA silencing"[tiab:~1] OR "posttranscriptional gene silencing"[tiab] OR "post transcriptional gene silencing"[tiab] OR "chimeric antigen receptor therapy"[tiab:~1] OR "chimeric antigen receptor therapies"[tiab:~1] OR "CAR-T cell therap*"[tiab] OR "CAR Tcell therap*"[tiab] OR CAR-T[tiab] OR "chimeric antigen receptor T-cell"[tiab] OR "adoptive immunotherap*"[tiab] OR "adoptive immuno therapy"[tiab:~2] OR "adoptive immuno therapies"[tiab:~2] OR "autologous cell therap*"[tiab] OR "autologous cell immunotherap*"[tiab] OR "IGV 001"[tiab] OR "IGV001"[tiab] OR "antisense oligonucleotide*"[tiab] OR "anti sense oligonucleotide*"[tiab] OR Goldspire[tiab] OR "zinc finger nuclease*" OR ZFN[tiab] OR ("patient derived"[tiab] AND "gene therap*"[tiab] AND cellular[tiab]) OR "Receptors, Chimeric Antigen"[mh] OR "Immunotherapy, Adoptive"[mh] OR "RNA Interference"[mh] OR "RNAi Therapeutics"[mh] OR "Oligonucleotides, Antisense"[tiab] OR "Zinc Finger Nucleases"[mh]	199 194
5	#1 OR #2 OR #3 OR #4	300 468
6	"trial protocol"[ti] OR "study protocol"[ti] OR Clinical Trial Protocol[pt]	30 376
7	#5 NOT #6	300 436
8	rodent*[tiab] OR mouse[tiab] OR mice[tiab] OR animal*[tiab] OR pig[tiab] OR pigs[tiab] OR porcine*[tiab] OR "non human*"[tiab] OR "nonhuman*"[tiab]	3 000 069
9	#7 NOT #8	223 389
10	"clinical trial"[pt] OR "randomized controlled trial"[pt] OR "controlled clinical trial"[pt] OR "Clinical Study" [pt:NoExp] OR "randomized controlled trials as topic"[mh] OR "Double-Blind Method"[mh] OR "randomized controlled trial*"[tiab] OR (randomized[ti] AND controlled[ti] AND trial[ti]) OR "randomized trial*"[tiab] OR "randomised controlled trial*"[tiab] OR (randomised[ti] AND controlled[ti] AND trial[ti]) OR "randomised trial*"[ti] OR "clinical trial*"[ti] OR "double blind*"[ti] OR "clinical stud*"[ti] OR "placebo controlled trial*"[tiab] OR "systematic review"[pt] OR "Systematic Reviews as Topic"[mh] OR "systematic review*"[ti] OR "review of literature"[ti] OR "rapid review*"[ti] OR "rapid	1 835 671

	evidence assessment*"[ti] OR "scoping review*"[ti] OR "mapping review*"[ti] OR "literature review*"[ti] OR "Pragmatic Clinical Trial"[pt] OR "Clinical Trial, Phase IV"[pt] OR "Clinical Trial, Phase III"[pt] OR "Clinical Trial, Phase II"[pt] OR "Clinical Trial, Phase I"[pt]	
11	#9 AND #10	4336
12	#11 AND (2018/11/1:**2025**/12/31[pdat])	1914
13	#12 AND ((humans[Filter]) AND (english[Filter]))	**1541**
	Total (after deduplication)	**1538**

	**PubMed – Humans; English limits applied**	
#1	(gene-editing[tiab] OR "gene editing"[tiab]) AND (therap*[tiab] OR treatment*[tiab])	7,340
#2	"gene therapy"[tiab] OR "gene therapies"[tiab] OR "gene engineering"[tiab] OR "gene transfer*"[tiab]	99,731
#3	"Gene Editing/ethics"[mh] OR "Gene Editing/instrumentation"[mh] OR "Gene Editing/legislation and jurisprudence"[mh] OR "Gene Editing/standards"[mh] OR "Gene Editing/statistics and numerical data"[mh] OR "Gene Editing/trends"[mh] OR "Genetic Therapy/adverse effects"[mh] OR "Genetic Therapy/classification"[mh] OR "Genetic Therapy/epidemiology"[mh] OR "Genetic Therapy/ethics"[mh] OR "Genetic Therapy/instrumentation"[mh] OR "Genetic Therapy/legislation and jurisprudence"[mh] OR "Genetic Therapy/methods"[mh] OR "Genetic Therapy/mortality"[mh] OR "Genetic Therapy/nursing"[mh] OR "Genetic Therapy/psychology"[mh] OR "Genetic Therapy/standards"[mh] OR "Genetic Therapy/statistics and numerical data"[mh] OR "Genetic Therapy/therapeutic use"[mh] OR "Genetic Therapy/therapy"[mh] OR "Genetic Therapy/trends"[mh] OR "Gene Transfer Techniques/adverse effects"[mh] OR "Gene Transfer Techniques/drug effects"[mh] OR "Gene Transfer Techniques/immunology"[mh] OR "Gene Transfer Techniques/therapy"[mh] OR "Gene Transfer, Horizontal/adverse effects"[mh] OR "Gene Transfer, Horizontal/drug effects"[mh] OR "Gene Transfer, Horizontal/immunology"[mh] OR "Gene Transfer, Horizontal/therapeutic use"[mh]	32,152
#4	"betibeglogene autotemcel"[tiab] OR zynteglo[tiab] OR "elivaldogene autotemcel"[tiab] OR skysona[tiab] OR "adeno associated virus"[tiab] OR "adenoassociated virus"[tiab] OR "adeno-associated virus"[tiab] OR CRISPR*[tiab] OR "clustered regularly interspaced short palindromic repeats"[tiab] OR "Clustered Regularly Interspaced Short Palindromic Repeats"[Mesh] OR "betibeglogene autotemcel" [Supplementary Concept]	75,258
#5	#1 OR #2 OR #3 OR #4	177,496
#6	"trial protocol"[ti] OR "study protocol"[ti] OR "Clinical Trial Protocol"[pt]	36,903
#7	#5 NOT #6	177,475

#8	rodent*[tiab] OR rat[tiab] OR rats[tiab] OR mouse[tiab] OR mice[tiab] OR animal*[tiab] OR pig[tiab] OR pigs[tiab] OR porcine*[tiab] OR "non human*"[tiab] OR "nonhuman*"[tiab]	4,231,084
#9	#7 NOT #8	120,741
#10	"clinical trial"[pt] OR "randomized controlled trial"[pt] OR "controlled clinical trial"[pt] OR "Clinical Study" [pt:NoExp] OR "randomized controlled trials as topic"[mh] OR "Double-Blind Method"[mh] OR "randomized controlled trial*"[tiab] OR (randomized[ti] AND controlled[ti] AND trial[ti]) OR "randomized trial*"[tiab] OR "randomised controlled trial*"[tiab] OR (randomised[ti] AND controlled[ti] AND trial[ti]) OR "randomised trial*"[ti] OR "clinical trial*"[ti] OR "double blind*"[ti] OR "clinical stud*"[ti] OR "placebo controlled trial*"[tiab] OR "systematic review"[pt] OR "Systematic Reviews as Topic"[mh] OR "systematic review*"[ti] OR "review of literature"[ti] OR "rapid review*"[ti] OR "rapid evidence assessment*"[ti] OR "scoping review*"[ti] OR "mapping review*"[ti] OR "literature review*"[ti] OR "Pragmatic Clinical Trial"[pt] OR "Clinical Trial, Phase IV"[pt] OR "Clinical Trial, Phase III"[pt] OR "Clinical Trial, Phase II"[pt] OR "Clinical Trial, Phase I"[pt]	1,950,126
#11	#9 AND #10	2,235
#12	(**2018/11/01:2025/03/31[pdat]**)	9,772,221
#12	(#11 AND #12) AND ((humans[Filter]) AND (english[Filter]))	**640**
	Total (Post-deduplication)	**151**

Section 2. Searches of the Embase Database

	**Embase–** Limits applied: ([cochrane review]/lim OR [systematic review]/lim OR [meta analysis]/lim OR [controlled clinical trial]/lim OR [randomized controlled trial]/lim) AND ([article]/lim OR [article in press]/lim OR [data papers]/lim OR [review]/lim) AND [english]/lim AND [humans]/lim AND [embase]/lim AND [01-11-2018]/sd NOT [01-01-2025]/sd	
#1	(gene-editing OR "gene editing"):ab,ti AND (therap* OR treatment*):ab,ti	37
#2	('gene therapy' OR 'gene therapies' OR 'gene engineering' OR 'gene transfer'):ab,ti	711
#3	('betibeglogene autotemcel' OR zynteglo OR 'elivaldogene autotemcel' OR skysona OR 'adeno associated virus' OR 'adenoassociated virus' OR 'adeno-associated virus' OR CRISPR* OR 'clustered regularly interspaced short palindromic repeats'):ab,ti	340
#4	'horizontal gene transfer'/mj OR 'gene editing'/mj OR 'gene therapy'/mj	144
#5	#1 OR #2 OR #3 OR #4	379
#6	("trial protocol"):ti OR ("study protocol"):ti	22,320
#7	#5 NOT #6	**376**
#8	(rodent* OR mouse OR mice OR animal* OR pig OR pigs OR porcine* OR "non human*" OR "nonhuman*"):ab,ti	**3,643,044**
#9	#7 NOT #8	**323**
	Total (Post-deduplication)	**40**

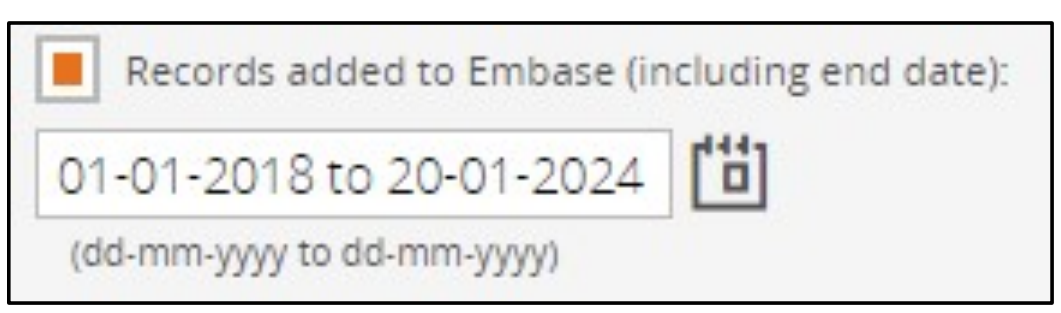

No.	Embase	Results
	**Embase:** Limits applied: ([cochrane review]/lim OR [systematic review]/lim OR [meta analysis]/lim OR [controlled clinical trial]/lim OR [randomized controlled trial]/lim) AND ([article]/lim OR [article in press]/lim OR [data papers]/lim OR [review]/lim) AND [english]/lim AND [humans]/lim AND [embase]/lim AND [01-11-2018]/sd NOT [01-01-**2026**]/sd	
1	(gene-editing OR "gene editing"):ab,ti AND (therap* OR treatment*):ab,ti	49
2	('gene therapy' OR 'gene therapies' OR 'gene engineering' OR 'gene transfer'):ab,ti	377
3	('betibeglogene autotemcel' OR zynteglo OR 'elivaldogene autotemcel' OR skysona OR 'adeno associated virus' OR 'adenoassociated virus' OR 'adeno-associated virus' OR CRISPR* OR 'clustered regularly interspaced short palindromic repeats'):ab,ti	165
4	'horizontal gene transfer'/mj OR 'gene editing'/mj OR 'gene therapy'/mj	156

5	#1 OR #2 OR #3 OR #4	524
6	("trial protocol"):ti OR ("study protocol"):ti	24 147
7	#5 NOT #6	**521**
8	(rodent* OR mouse OR mice OR animal* OR pig OR pigs OR porcine* OR "non human*" OR "nonhuman*"):ab,ti	**3 744 573**
9	#7 NOT #8	**448**
	Total (after deduplication)	**298**

	**Embase–** Limits applied: ([cochrane review]/lim OR [systematic review]/lim OR [meta analysis]/lim OR [controlled clinical trial]/lim OR [randomized controlled trial]/lim) AND ([article]/lim OR [article in press]/lim OR [data papers]/lim OR [review]/lim) AND [english]/lim AND [humans]/lim AND [embase]/lim AND [**2018-10-31 to 2025-04-01**]/pd	
#1	(gene-editing OR "gene editing"):ab,ti AND (therap* OR treatment*):ab,ti	10,419
#2	('gene therapy' OR 'gene therapies' OR 'gene engineering' OR 'gene transfer'):ab,ti	125,012
#3	('betibeglogene autotemcel' OR zynteglo OR 'elivaldogene autotemcel' OR skysona OR 'adeno associated virus' OR 'adenoassociated virus' OR 'adeno-associated virus' OR CRISPR* OR 'clustered regularly interspaced short palindromic repeats'):ab,ti	101,947
#4	'horizontal gene transfer'/mj OR 'gene editing'/mj OR 'gene therapy'/mj	50,388
#5	#1 OR #2 OR #3 OR #4	227,114
#6	("trial protocol"):ti OR ("study protocol"):ti	28,484
#7	#5 NOT #6	**227,171**
#8	(rodent* OR rat OR rats OR mouse OR mice OR animal* OR pig OR pigs OR porcine* OR "non human*" OR "nonhuman*"):ab,ti	5,252,285
#9	(#7 NOT #8) AND ([cochrane review]/lim OR [systematic review]/lim OR [meta analysis]/lim OR [controlled clinical trial]/lim OR [randomized controlled trial]/lim) AND ('article'/it OR 'article in press'/it OR 'data papers'/it) AND [english]/lim AND [embase]/lim AND [**2018-10-31 to 2025-04-01**]/pd	**234**
	Total (Post-deduplication)	**92**

Section 3. Searches of the Web of Science Database

	**Web of Science (Science Citation Index Expanded (SCI-EXPANDED), Social Sciences Citation Index (SSCI), Arts & Humanities Citation Index (A&HCI), Emerging Sources Citation Index (ESCI):– index date range: 11/1/2018-12/31/2024**	
#1	(TI=(gene-editing) OR TI=("gene editing")) AND (TI=(therap*) OR TI=(treatment*)) OR (TI=(gene-editing) OR TI=("gene editing")) AND (AB=(therap*) OR AB=(treatment*)) OR (AB=(gene-editing) OR AB=("gene editing")) AND (TI=(therap*) OR TI=(treatment*)) OR (AB=(gene-editing) OR AB=("gene editing")) AND (AB=(therap*) OR AB=(treatment*))	4,343
#2	TI=("gene therapy" OR "gene therapies" OR "gene engineering" OR "gene transfer") OR AB=("gene therapy" OR "gene therapies" OR "gene engineering" OR "gene transfer")	99,009
#3	TI=("betibeglogene autotemcel" OR zynteglo OR "elivaldogene autotemcel" OR skysona OR "adeno associated virus" OR "adenoassociated virus" OR "adeno-associated virus" OR CRISPR* OR "clustered regularly interspaced short palindromic repeats") OR AB=("betibeglogene autotemcel" OR zynteglo OR "elivaldogene autotemcel" OR skysona OR "adeno associated virus" OR "adenoassociated virus" OR "adeno-associated virus" OR CRISPR* OR "clustered regularly interspaced short palindromic repeats")	56,544
#4	#1 OR #2 OR #3	149,783
#5	TS=(rat OR rats OR monkey OR monkeys OR primates OR primate OR macaque* OR mouse OR mice OR dog OR canine OR canines OR rabbit OR rabbits OR porcine)	4,110,408
#6	#4 NOT #5	102,611
#7	TI=("trial protocol") OR TI=("study protocol")	20,431
#8	#6 NOT #7	102,605
#9	TI=("randomized controlled trial") OR TI=("randomised controlled trial") OR TI=(RCT) OR TI=("controlled clinical trial") OR TI=("clinical trial") OR TI=("clinical study") OR TI=("systematic review*") OR TI=("scoping review*") OR TI=("review of literature") OR TI=("literature review") OR TI=("mapping review*") OR TI=("rapid evidence assessment*") OR AK=("randomized controlled trial") OR AK=("randomised controlled trial") OR AK=(RCT) OR AK=("controlled clinical trial") OR AK=("clinical trial") OR AK=("clinical study") OR AK=("systematic review*") OR AK=("scoping review*") OR AK=("review of literature") OR AK=("literature review") OR AK=("mapping review*") OR AK=("rapid evidence assessment*") OR KP=("randomized controlled trial") OR KP=("randomised controlled trial") OR KP=(RCT) OR KP=("controlled clinical trial") OR KP=("clinical trial") OR KP=("clinical study") OR KP=("systematic review*") OR KP=("scoping review*") OR KP=("review of literature")	762,810

	OR KP=("literature review") OR KP=("mapping review*") OR KP=("rapid evidence assessment*")	
#10	#8 AND #9	1,237
#11	#10 AND (DT==("REVIEW" OR "ARTICLE" OR "EARLY ACCESS") AND LA==("ENGLISH") AND DOP=(2018-11-01/2024-01-31))	**381**
	Total (Post-deduplication)	**229**

	**Web of Science (Science Citation Index Expanded (SCI-EXPANDED), Social Sciences Citation Index (SSCI), Arts & Humanities Citation Index (A&HCI), Emerging Sources Citation Index (ESCI):– index date range: 11/1/2018-01/20/2024**	
1	TI=(IMLYGIC OR "Talimogene laherparepvec" OR T-VEC OR LUXTURNA OR "Voretigene neparvovec" OR Zolgensma OR "Onasemnogene abeparvovec" OR CRISPR* OR "clustered regularly interspaced short palindromic repeats") OR AB=(IMLYGIC OR "Talimogene laherparepvec" OR T-VEC OR LUXTURNA OR "Voretigene neparvovec" OR Zolgensma OR "Onasemnogene abeparvovec" OR CRISPR* OR "clustered regularly interspaced short palindromic repeats")	45,601
2	TI=("trial protocol") OR TI=("study protocol")	20,431
3	#1 NOT #2	45,599
4	TS=(rat OR rats OR monkey OR monkeys OR primates OR primate OR macaque* OR mouse OR mice OR dog OR canine OR canines OR rabbit OR rabbits OR porcine)	4,110,408
5	#3 NOT #4	36,416
6	TI=("observational stud*" OR "cohort stud*" OR "cohort analys*" OR "incidence stud*" OR "follow up" OR followup OR "comparative stud*" OR "pre post" OR "before after" OR "longitudinal stud*" OR "longitudinal analys*" OR "prospective stud*" OR "retrospective stud*" OR questionnaire* OR survey OR "panel stud*") OR AB=("observational stud*" OR "cohort stud*" OR "cohort analys*" OR "incidence stud*" OR "follow up" OR followup OR "comparative stud*" OR "pre post" OR "before after" OR "longitudinal stud*" OR "longitudinal analys*" OR "prospective stud*" OR "retrospective stud*" OR questionnaire* OR survey OR "panel stud*")	4,215,401
7	#5 AND #6	508
8	#7 AND (DT==("REVIEW" OR "ARTICLE" OR "EARLY ACCESS") AND LA==("ENGLISH")) AND DOP=(2018-11-01/2024-01-31)	**371**
	Total (Post-deduplication)	**203**

No.	Web of Science	Results
	**Web of Science** (Science Citation Index Expanded (SCI-EXPANDED), Social Sciences Citation Index (SSCI), Arts & Humanities Citation Index (A&HCI), Emerging Sources Citation Index (ESCI)– index date range: 11/1/2018-12/31/2025	

1	(TI=(gene-editing) OR TI=("gene editing")) AND (TI=(therap*) OR TI=(treatment*)) OR (TI=(gene-editing) OR TI=("gene editing")) AND (AB=(therap*) OR AB=(treatment*)) OR (AB=(gene-editing) OR AB=("gene editing")) AND (TI=(therap*) OR TI=(treatment*)) OR (AB=(gene-editing) OR AB=("gene editing")) AND (AB=(therap*) OR AB=(treatment*))	5439
2	TI=("gene therapy" OR "gene therapies" OR "gene engineering" OR "gene transfer") OR AB=("gene therapy" OR "gene therapies" OR "gene engineering" OR "gene transfer")	102 489
3	TI=("betibeglogene autotemcel" OR zynteglo OR "elivaldogene autotemcel" OR skysona OR "adeno associated virus" OR "adenoassociated virus" OR "adeno-associated virus" OR CRISPR* OR "clustered regularly interspaced short palindromic repeats") OR AB=("betibeglogeneautotemcel" OR zynteglo OR "elivaldogene autotemcel" OR skysona OR "adeno associated virus" OR "adenoassociated virus" OR "adeno-associated virus" OR CRISPR* OR "clustered regularly interspaced short palindromic repeats")	65 491
4	#1 OR #2 OR #3	161 864
5	TS=(rat OR rats OR monkey OR monkeys OR primates OR primate OR macaque* OR mouse OR mice OR dog OR canine OR canines OR rabbit OR rabbits OR porcine)	4 350 911
6	#4 NOT #5	111 763
7	TI=("trial protocol") OR TI=("study protocol")	23 474
8	#6 NOT #7	111 757
9	TI=("randomized controlled trial") OR TI=("randomised controlled trial") OR TI=(RCT) OR TI=("controlled clinical trial") OR TI=("clinical trial") OR TI=("clinical study") OR TI=("systematic review*") OR TI=("scoping review*") OR TI=("review of literature") OR TI=("literature review") OR TI=("mapping review*") OR TI=("rapid evidence assessment*") OR AK=("randomized controlled trial") OR AK=("randomised controlled trial") OR AK=(RCT) OR AK=("controlled clinical trial") OR AK=("clinical trial") OR AK=("clinical study") OR AK=("systematic review*") OR AK=("scoping review*") OR AK=("review of literature") OR AK=("literature review") OR AK=("mapping review*") OR AK=("rapid evidence assessment*") OR KP=("randomized controlled trial") OR KP=("randomised controlled trial") OR KP=(RCT) OR KP=("controlled clinical trial") OR KP=("clinical trial") OR KP=("clinical study") OR KP=("systematic review*") OR KP=("scoping review*") OR KP=("review of literature") OR KP=("literature review") OR KP=("mapping review*") OR KP=("rapid evidence assessment*")	848 771
10	#8 AND #9	1374
11	#10 AND (DT==("REVIEW" OR "ARTICLE" OR "EARLY ACCESS") AND LA==("ENGLISH") AND DOP=(2018-11-01/**2026**-01-31))	**489**
	Total (after deduplication)	**301**

	**Web of Science** (Science Citation Index Expanded (SCI-EXPANDED), Social Sciences Citation Index (SSCI), Arts & Humanities Citation Index (A&HCI), Emerging Sources Citation Index (ESCI):– index date range: **11/1/2018-03/31/2025**	
#1	TI=((gene-editing OR "gene editing") AND (therap* OR treatment*)) OR AB=((gene-editing OR "gene editing") AND (therap* OR treatment*))	6,660
#2	TI=("gene therapy" OR "gene therapies" OR "gene engineering" OR "gene transfer") OR AB=("gene therapy" OR "gene therapies" OR "gene engineering" OR "gene transfer")	108,001
#3	TI=("betibeglogene autotemcel" OR zynteglo OR "elivaldogene autotemcel" OR skysona OR "adeno associated virus" OR "adenoassociated virus" OR "adeno-associated virus" OR CRISPR* OR "clustered regularly interspaced short palindromic repeats") OR AB=("betibeglogene autotemcel" OR zynteglo OR "elivaldogene autotemcel" OR skysona OR "adeno associated virus" OR "adenoassociated virus" OR "adeno-associated virus" OR CRISPR* OR "clustered regularly interspaced short palindromic repeats")	76,024
#4	#1 OR #2 OR #3	177,541
#5	TS=(rat OR rats OR rodent* OR monkey OR monkeys OR primates OR primate OR macaque* OR mouse OR mice OR dog OR canine OR canines OR rabbit OR rabbits OR porcine)	4,537,757
#6	#4 NOT #5	123,966
#7	TI=("trial protocol") OR TI=("study protocol")	26,341
#8	#6 NOT #7	123,960
#9	TI=("randomized controlled trial") OR TI=("randomised controlled trial") OR TI=(RCT) OR TI=("controlled clinical trial") OR TI=("clinical trial") OR TI=("clinical study") OR TI=("systematic review*") OR TI=("scoping review*") OR TI=("review of literature") OR TI=("literature review") OR TI=("mapping review*") OR TI=("rapid evidence assessment*") OR AK=("randomized controlled trial") OR AK=("randomised controlled trial") OR AK=(RCT) OR AK=("controlled clinical trial") OR AK=("clinical trial") OR AK=("clinical study") OR AK=("systematic review*") OR AK=("scoping review*") OR AK=("review of literature") OR AK=("literature review") OR AK=("mapping review*") OR AK=("rapid evidence assessment*") OR KP=("randomized controlled trial") OR KP=("randomised controlled trial") OR KP=(RCT) OR KP=("controlled clinical trial") OR KP=("clinical trial") OR KP=("clinical study") OR KP=("systematic review*") OR KP=("scoping review*") OR KP=("review of literature") OR KP=("literature review") OR KP=("mapping review*") OR KP=("rapid evidence assessment*")	944,007
#10	#8 AND #9	1,546
#11	#10 AND (DT==("REVIEW" OR "ARTICLE" OR "EARLY ACCESS") AND LA==("ENGLISH") AND DOP=(**2018-11-01/2025-03-31**))	**496**
	Total (Post-deduplication)	**15**

Section 4. Searches of the ClinicalTrials.gov Database

Set #	**ClinicalTrials.gov Limits:** Year range: 2018-2024; Study Status: Active_Not Recruiting; Study Type: Interventional
**#1**	(gene-editing OR "gene editing") AND (therap* OR treatment*)
**#2**	"gene therapy" OR "gene therapies" OR "gene engineering" OR "gene transfer*"
**#3**	"betibeglogene autotemcel" OR zynteglo OR "elivaldogene autotemcel" OR skysona OR "adeno associated virus" OR "adenoassociated virus" OR "adeno-associated virus" OR CRISPR* OR "clustered regularly interspaced short palindromic repeats"
**#4**	Total # of results = **92**

Set #	**ClinicalTrials.gov Limits:** Year range: 2018-2024; Study Status: Active_Not Recruiting; Study Type: Interventional
**#1**	Lenmeldy OR olezarsen OR Vupanorsen OR IONIS-APOCIII-LRx OR AKCEA-APOCIII-LRX OR Libmeldy OR "atidarsagene autotemcel" OR "Beqvez" OR "Fidanacogene elaparvovec" OR "Fidanacogene elaparvovec-dzkt"
**#2**	Total # of results = **5**
