# Supplementary material for: Evaluating selected gene therapies for cardiovascular diseases: an updated scoping review and evidence map of randomised controlled trials
Source: Open Heart. 2026 Oct 20;13(2):e004374. doi: 10.1136/openhrt-2026-004374 (PMC13711567; doi:10.1136/openhrt-2026-004374)
Supplement: Supplementary file 2 [file openhrt-13-2-s002.pdf]

Supplementary Material 2: Evidence Table (n=28)

Author, Year RefID	Intervention Category	Indication<br>Administration Frequency	Outcomes Measured<br>Sample (N)<br>Length of Follow-up	Population Age<br>Treatment Setting<br>Race/Ethnicity<br>Percent Female<br>Comorbidities	Effect	Serious Adverse Events/Harms<br><br>Intervention SAEs / Intervention Arm N
Indication: Dyslipidemia						
**Ballantyne, 2024**[1]	RNAi	Indication: Mixed hyperlipidemia<br>Administration frequency: limited multiple;<br>quarterly or half-yearly	Outcomes: Triglycerides, APOC3, non-HDL cholesterol, apoB, LDL cholesterol, remnant cholesterol, lipo-protein(a), high-sensitivity C-reactive protein<br><br>N=353<br><br>Follow-up: 6 months	Population age: NR<br>Treatment setting: Unclear; 36 sites in multiple countries<br>Race/Ethnicity: W<br>Female: 45%<br>Comorbidities: Diabetes, chronic kidney disease	Improvement	Serious adverse events: Intervention: 19; Control: 5<br><br>7.1%
**Bergmark, 2022**[2]	Antisense	Indication: Statin-treated patients with elevated cholesterol<br>Administration frequency: limited multiple	Outcomes: Non-high-density lipoprotein cholesterol, triglycerides, low-density lipoprotein cholesterol , ApoB, ANGPTL3<br><br>N=286<br><br>Follow-up: 6 months	Population age: NR<br>Treatment setting: Unclear; multiple institutions<br>Race/Ethnicity: Asian, Black, White<br>Female: 44%<br>Comorbidities: Type 2 diabetes; previous myocardial infarction; previous resvascularization; previous stroke; peripheral artery disease	Improvement	Treatment-emergent serious adverse events: Intervention: 15; Control: 4<br><br>9.6%

Author, Year RefID	Intervention Category	Indication<br>Administration Frequency	Outcomes Measured<br>Sample (N)<br>Length of Follow-up	Population Age<br>Treatment Setting<br>Race/Ethnicity<br>Percent Female<br>Comorbidities	Effect	Serious Adverse Events/Harms<br><br>Intervention SAEs / Intervention Arm N
**Bergmark, 2024**[3]	Antisense	Indication: Hypertriglyceridemia in patients at high cardiovascular risk<br>Administration frequency: limited multiple	Outcomes: Triglyceride levels, APOC3, very-low-density lipoprotein cholesterol, total apolipoprotein B, non–high-density lipoprotein cholesterol, low-density lipoprotein cholesterol , moderate hypertriglyceridemia, pancreatitis events<br><br>N=154<br><br>Follow-up:12 months	Population age: NR<br>Treatment setting: Unclear; multiple institutions<br>Race/Ethnicity: Asian, Black, Hispanic/Latino (H/L), White<br>Female: 42%<br>Comorbidities: Pancreatitis; atherosclerotic cardiovascular disease; diabetes mellitus; chronic kidney disease	Sustained improvement	Serious adverse events: Intervention: 11; Placebo: 2<br><br>6.2%
**Fukuhara, 2023**[4]	Antisense	Indication: Dyslipidemia, elevated triglycerides<br>Administration frequency: limited multiple; single ascending dose	Outcomes: Angiopoietin-like 3, apolipoprotein, high-density lipoprotein cholesterol, low-density lipoprotein cholesterol, triglycerides, safety<br><br>N=12<br><br>Follow-up: 3 months	Population age: 33-63<br>Treatment setting: TMC<br>Race/Ethnicity: Asian<br>Female: 0%<br>Comorbidities: NR	Improvement	No serious adverse events.<br><br>0%
**Gaudet, 2024**[5]	RNAi	Indication: Severe hypertriglyceridemia<br>Administration frequency: limited multiple; 2 doses	Outcomes: Change in triglyceride levels<br><br>N=226<br><br>Follow-up: 11 months	Population age: 55 (mean)<br>Treatment setting: TMC<br>Race/Ethnicity: Alaskan Native and American Indian, Asian, Black,	Improvement	Serious treatment emergent adverse events: Intervention: 13; Placebo: 10<br><br>7.8%

**Author, Year RefID**	**Intervention Category**	**Indication Administration Frequency**	**Outcomes Measured Sample (N) Length of Follow-up**	**Population Age Treatment Setting Race/Ethnicity Percent Female Comorbidities**	**Effect**	**Serious Adverse Events/Harms<br><br>Intervention SAEs / Intervention Arm N**
				Hispanic/Latino, Native Hawaiian and other Pacific Islander, White<br>Female: 22%<br>Comorbidities: NR		
**Luo, 2023[6]**	RNAi	Indication: Hypercholesterolemia<br>Administration frequency: once	Outcomes: Total cholesterol, triglycerides, high-density lipoprotein, cholesterol, calculated LDL-C, PCSK9<br><br>N=40<br><br>Follow-up: 3 months	Population age: NR<br>Treatment setting: Unclear; multiple insitutions<br>Race/Ethnicity: NR<br>Female: 73%<br>Comorbidities: NR	Improvement	No serious adverse events.<br><br>0%
**Nissen, 2022[7]**	RNAi	Indication: Elevated plasma lipoprotein(a)<br>Administration frequency: once	Outcomes: Plasma Lp(a) concentrations, low-density lipoprotein, high-density lipoprotein, total cholesterol, triglycerides, apolipoprotein B, oxidized low-density lipoprotein, interleukin 6, C-reactive protein<br><br>N=33<br><br>Follow-up: 5 months	Population age: NR<br>Treatment setting: Unclear; multiple institutions<br>Race/Ethnicity: Asian, Black, White<br>Female: 53%<br>Comorbidities: NR	Improvement	Serious adverse events: Intervention: 1; Placebo: 0<br><br>4.2%
**Raal, 2024[8]**	RNAi	Indication: Homozygous familial hypercholesterolemia	Outcomes: LDL-C, non-HDL-C-lipoprotein and total cholesterol<br><br>N=56	Population age: NR<br>Treatment setting: TMC<br>Race/Ethnicity: NR<br>Female: 60%	No improvement	Tretment-emergent serious adverse events: Intervention: 10; Placebo: 4<br><br>27.0%

Author, Year RefID	Intervention Category	Indication<br>Administration Frequency	Outcomes Measured<br>Sample (N)<br>Length of Follow-up	Population Age<br>Treatment Setting<br>Race/Ethnicity<br>Percent Female<br>Comorbidities	Effect	Serious Adverse Events/Harms<br><br>Intervention SAEs / Intervention Arm N
		Administration frequency: limited multiple	Follow-up: 24 months	Comorbidities: NNR		
**Rekić, 2022**[9]	Antisense	Indication: Hypercholesterolaemia<br>Administration frequency: limited multiple	Outcomes: Heart rate, QT prolongation<br><br>N=73<br><br>Follow-up: 4 months	Population age: NR<br>Treatment setting:<br>Race/Ethnicity: Asian, White<br>Female: 0%<br>Comorbidities: NR	Not reported	Serious adverse events related to QT prolongation:<br>Intervention: 0<br>No control group<br><br>0%
**Rosenson, 2024**[10]	RNAi	Indication: Mixed hyperlipidemia<br>Administration frequency: limited multiple; day 1 and week 12	Outcomes: Change in triglyceride levels, safety<br><br>N=204<br><br>Follow-up: 9 months	Population age: 48-74<br>Treatment setting: NR<br>Race/Ethnicity: White, Other<br>Female: 47%<br>Comorbidities: Chronic kidney disease, type 2 diabetes	Improvement	Serious adverse events:<br>Intervention: 6; Placebo: 4<br><br>3.9%
**Tardif, 2022**[11]	Antisense	Indication: Hypertriglyceridaemia and high cardiovascular risk<br>Administration frequency: limited multiple; monthly dosage for at least 6 months	Outcomes: Change in triglyceride levels, change in cholesterol levels<br><br>N=114<br><br>Follow-up: 3 months	Population age: 57-72<br>Treatment setting:<br>Race/Ethnicity: White, Other<br>Female: 14%<br>Comorbidities: Coronary artery disease, coronary revascularization, acute myocardial infarction, premature CAD, carotid artery	Improvement	Serious adverse events:<br>Intervention: 9; Placebo: 1<br><br>10.0%

Author, Year RefID	Intervention Category	Indication Administration Frequency	Outcomes Measured Sample (N) Length of Follow-up	Population Age Treatment Setting Race/Ethnicity Percent Female Comorbidities	Effect	Serious Adverse Events/Harms<br><br>Intervention SAEs / Intervention Arm N
				disease, peripheral artery disease, stroke, transient ischaemic attack, heart failure, type 2 diabetes		
**Zimerman, 2024**[12]	Antisense	Indication: High cholesterol<br>Administration frequency: limited multiple	Outcomes: Non-high density lipoprotein cholesterol, very low-density lipoprotein cholesterol, remnant cholesterol<br><br>N=286<br><br>Follow-up: 6 months	Population age: NR<br>Treatment setting:<br>Race/Ethnicity: Asian, Black, White<br>Female: 44%<br>Comorbidities: NR	Improvement	Adverse events not measured.<br><br>NR
Indication: Ischemia						
**Barć, 2020**[13]	Plasmid-based	Indication: Critical limb ischemia complicated by diabetes mellitus<br>Administration frequency: once	Outcomes: Efficacy, ankle-brachial index, pain,VEGF expression<br><br>N=28<br><br>Follow-up: 3 months	Population age: 40-85<br>Treatment Setting: Other<br>Race/Ethnicity: NR<br>Female: 32%<br>Comorbidities: NR	Improvement	Serious complications: Intervention: 0; Control: 0<br><br>0%
**Gu, 2021**[14] **RCT**	Plasmid-based	Indication: Critical limb ischemia<br>Administration frequency: limited multiple	Outcomes: Pain relief, ulcer healing<br><br>N=240<br><br>Follow-up: 6 months	Population age: ~55 (mean)<br>Treatment Setting: Other, NR<br>Race/Ethnicity: Asian<br>Female: 13%<br>Comorbidities: None	Improvement	Serious adverse events: Intervention: 15; Placebo: 6<br><br>8.5%

Author, Year RefID	Intervention Category	Indication Administration Frequency	Outcomes Measured Sample (N) Length of Follow-up	Population Age Treatment Setting Race/Ethnicity Percent Female Comorbidities	Effect	Serious Adverse Events/Harms<br><br>Intervention SAEs / Intervention Arm N
**Gu, 2019[15] RCT**	Plasmid-based	Indication: Critical limb ischemia<br>Administration frequency: limited multiple	Outcomes: Safety, tolerability, efficacy<br><br>N=200<br><br>Follow-up: 6 months	Population age: 43-73<br>Treatment Setting: Other, multi-center<br>Race/Ethnicity: Asian<br>Female: 15%<br>Comorbidities: Hyperlipidemia, hypertension, diabetes	Improvement	Serious adverse events:<br>Intervention: 0; Placebo: 0<br><br>0%
**Hammad, 2020[16]**	Plasmid-based	Indication: Peripheral artery disease-related chronic limb-threatening ischemia<br>Administration frequency: once	Outcomes: Efficacy, wound healing improvement, safety<br><br>N=109<br><br>Follow-up: 6 months	Population age: 56-83<br>Treatment Setting: 21 sites in US<br>Race/Ethnicity: NR<br>Female: 59%<br>Comorbidities: End stage renal disease, diabetes	No improvement	Major adverse limb events and deaths:<br>Intervention: 17; Placebo: 11<br><br>24.3%
Indication: Transthyretin amyloidosis						
**Fontana, 2025[17]**	RNAi	Indication: Transthyretin amyloidosis with cardiomyopathy<br>Administration frequency: limited multiple; every 12 weeks for up to 36 months	Outcomes: Death from any cause and reccurent cardiovascular events, change in functional capacity, safety<br><br>N=655<br><br>Follow-up: 36 months	Population age: 45-85<br>Treatment setting: Unclear; global study<br>Race/Ethnicity: Asian, Black, White<br>Female: 8%<br>Comorbidities: NR	Sustained improvement	Serious adverse events:<br>Intervention: 201; Placebo: 220<br><br>61.7%
**Garcia-Pavia, 2024[18]**	RNAi	Indication: Hereditary transthyretin-mediated amyloidosis with polyneuropathy	Outcomes: Cardiac symptom change measures; cardiac safety events	Population age: 26-85<br>Treatment setting:	Sustained improvement	Cardiac serious adverse events:<br>Intervention: 11; Placebo: 10<br><br>9.0%

Author, Year RefID	Intervention Category	Indication<br>Administration Frequency	Outcomes Measured<br>Sample (N)<br>Length of Follow-up	Population Age<br>Treatment Setting<br>Race/Ethnicity<br>Percent Female<br>Comorbidities	Effect	Serious Adverse Events/Harms<br><br>Intervention SAEs / Intervention Arm N
		Administration frequency: limited multiple; every 3 months for 18 months	N=199<br><br>Follow-up: 18 months	Race/Ethnicity: Asian, Black, White<br>Female: 28%<br>Comorbidities: NR		
**Judge, 2020**[19]	RNAi	Indication: Hereditary transthyretin-mediated (hATTR) amyliodosis with cardiomyopathy<br>Administration frequency:<br>limited multiple; daily for 5 days during first week, then weekly for remaining study	Outcomes: Change in 6MWT (m), percentage reduction in serum TTR levels, CV mortality, CV hospitalization, all-cause mortality<br><br>N=206<br><br>Follow-up: 7 months	Population age: 18-90<br>Treatment setting: TMC<br>Race/Ethnicity: Alaskan Native and American Indian, Black, White, Other<br>Female: 23%<br>Comorbidities:NR	No improvement	Severe adverse events: Intervention: 83; Placebo: 34<br><br>59.3%
**Maurer, 2023**[20]	RNAi	Indication: Transthyretin cardiac amyloidosis<br>Administration frequency: limited multiple; once every 3 weeks for 12 months	Outcomes: Change from baseline in the distance covered on the 6-minute walk test, change from baseline to month 12 in the Kansas City Cardiomyopathy Questionnaire–Overall Summary (KCCQ-OS) score, composite of death from any cause, cardiovascular events, hospitalizations for any cause, urgent heart failure visits<br><br>N=360<br><br>Follow-up: 12 months	Population age: 41-85 years<br>Treatment setting: TMC<br>Race/Ethnicity: Asian, Black, White, Other<br>Female: 11%<br>Comorbidities: Diabetes mellitus, Hypertension	Sustained improvement	Serious adverse events: Intervention: 61; Placebo: 63<br><br>33.7%

Author, Year RefID	Intervention Category	Indication Administration Frequency	Outcomes Measured Sample (N) Length of Follow-up	Population Age Treatment Setting Race/Ethnicity Percent Female Comorbidities	Effect	Serious Adverse Events/Harms<br><br>Intervention SAEs / Intervention Arm N
Indication: Heart failure						
**Lyon, 2020[21] RCT**	Adeno-associated virus (AAV)	Indication: Chronic heart failure supported with a left ventricular assist device<br>Administration frequency: once	Outcomes: Resting transthoracic echocardiography, exercise capacity, safety<br><br>N=5<br><br>Follow-up: 36 months	Population age: 29-69<br>Treatment Setting: CommHos<br>Race/Ethnicity: Black, White<br>Female: 20%<br>Comorbidities: NR	No improvement	No serious adverse events reported.<br><br>0%
**Taubel, 2020[22]**	Antisense	Indication: Chronic ischaemic heart failure<br>Administration frequency: limited multiple; twice, on days 1 and 28	Outcomes: QRS complex narrowing, safety<br><br>N=28<br><br>Follow-up: 4 months	Population age: 61-73 (mean year range)<br>Treatment setting: TMC<br>Race/Ethnicity: Asian, Black, White<br>Female: 4%<br>Comorbidities: Myocardial infarction, myocardial ischaemia, angina unstable, atrial fibrillation or flutter, hypertension, type 2 diabetes	Improvement	No serious adverse events reported.<br><br>0%
Indication: Other CVDs						
**Bakris, 2024[23]**	RNAi	Indication: Mild to moderate hypertension	Outcomes: Mean ambulatory systolic blood pressure, mean	Population age: 18-75	Improvement	No serious adverse events reported.

Author, Year RefID	Intervention Category	Indication Administration Frequency	Outcomes Measured Sample (N) Length of Follow-up	Population Age Treatment Setting Race/Ethnicity Percent Female Comorbidities	Effect	Serious Adverse Events/Harms<br><br>Intervention SAEs / Intervention Arm N
		Administration frequency: Limited multiple occasions; every 3-6 months for 6 months	ambulatory diastolic blood pressure, office blood pressure, serum angiotensinogen levels, daytime blood pressure, mighttime blood pressure<br><br>N=394<br><br>Follow-up: 6 months	Treatment setting: Unclear; 78 sites across 4 countries<br>Race/Ethnicity: Alaskan Native and American Indian (ANAI), Asian (AS), Black (B), Native Hawaiian and other Pacific Islander (NHPI), White (W)<br>Female: 44%<br>Comorbidities: Type II diabetes		0%
**Desai, 2023[24]**	RNAi	Indication: Hypertension<br>Administration frequency: once	Outcomes: Serum angiotensinogen levels, systolic and diastolic blood pressure<br><br>N=107<br><br>Follow-up: 6 months	Population age: 35-65<br>Treatment setting: Unclear; multiple institutions<br>Race/Ethnicity: Asian, Black, White<br>Female: 38%<br>Comorbidities: NR	Improvement	Serious adverse events: Intervention: 2; Placebo: 1<br><br>2.5%
**Gaudet, 2020[25]**	Antisense	Indication: Diabetes, hepatic steatosis, and hypertriglyceridaemia<br>Administration frequency: limited multiple	Outcomes: Triglyceride levels, ANGPTL3, total cholesterol (TC), LDL-C, HDL-C, VLDL cholesterol (VLDL-C), remnant cholesterol, non-HDL-C, polipoprotein (apo) B, ApoC-III, ApoAI, Lipoprotein(a), and FFAs, HbA1c,	Population age: 54 (mean)<br>Treatment setting:<br>Race/Ethnicity: Asian, Black, Hispanic/Latino, White<br>Female: 47%<br>Comorbidities: NR	Improvement	Serious adverse events: Intervention: 1; Placebo: 0<br><br>1.3%

**Author, Year RefID**	**Intervention Category**	**Indication Administration Frequency**	**Outcomes Measured Sample (N) Length of Follow-up**	**Population Age Treatment Setting Race/Ethnicity Percent Female Comorbidities**	**Effect**	**Serious Adverse Events/Harms<br><br>Intervention SAEs / Intervention Arm N**
			Homeostatic Model Assessment of Insulin Resistance (HOMA-IR), Fructosamine, Glycated albumin, HFF as assessed by MRIproton density fat fraction, Fatty liver index (FLI), Alanine aminotransferase (ALT), Aspartate aminotransferase (AST), high-sensitivity C-reactive protein<br><br>N=105<br><br>Follow-up: 11 months			
**Gouni-Berthold, 2021**[26]	Antisense	Indication: Multifactorial chylomicronaemia syndrome<br>Administration frequency: limited multiple	Outcomes: Change in triglyceride levels, response rate, safety<br><br>N=113<br><br>Follow-up: 9 months	Population age: 22-73<br>Treatment setting: Unclear; 38 clinical sites globally<br>Race/Ethnicity: Alaskan Native and American Indian, Asian, White<br>Female: 24%<br>Comorbidities: Pancreatitis, familial chlyomicronaemia syndrome, type 2 diabetes	Improvement	Serious adverse events: Intervention: 2; Placebo: 0<br><br>2.6%

Author, Year RefID	Intervention Category	Indication<br>Administration Frequency	Outcomes Measured<br>Sample (N)<br>Length of Follow-up	Population Age<br>Treatment Setting<br>Race/Ethnicity<br>Percent Female<br>Comorbidities	Effect	Serious Adverse Events/Harms<br><br>Intervention SAEs / Intervention Arm N
**Leikas, 2022**[27]	Adenovirus	Indication: Refractory angina<br>Administration frequency: once	Outcomes: Ventricular arrhythmias, heart rate variability, incidence of arrhythmias, incidence of new-onset arrhythmias, and pacemaker implantations<br><br>N=30<br><br>Follow-up: 97 months	Population age: 70<br>Treatment Setting: TAC<br>Race/Ethnicity: NR<br>Female: 23%<br>Comorbidities: NR	Not reported	Increase in serious arrythmias (including atrial fibrillation, ventricular tachycardia, and the use of a permanent pacemaker):<br>Intervention: 9; Placebo: 3<br><br>37.5%
**Leiter, 2019**[28]	RNAi	Indication: Atherosclerotic cardiovascular disease (ASCVD) or ASCVD-risk equivalents and elevated LDL-C<br>Administration frequency: limited multiple	Outcomes: LDL cholesterol, other lipid measures<br><br>N=501<br><br>Follow-up: 7 months	Population age: 18-≥65 years<br>Treatment setting: TMC<br>Race/Ethnicity: Alaskan Native and American Indian, Asian, Black, Hispanic/Latino, Native Hawaiian and other Pacific Islander, White, Other<br>Female: 35%<br>Comorbidities: Diabetes	Improvement	Serious adverse events:<br>Intervention: 41; Placebo: 9<br><br>Data pulled from original Ray, et al. (2017) trial.[29]<br><br>13.4%

Abbreviation: SAE = Serious Adverse Event.

References

1. Ballantyne CM, Vasas S, Azizad M, et al. Plozasiran, an RNA Interference Agent Targeting APOC3, for Mixed Hyperlipidemia. *N Engl J Med*. Sep 12 2024;391(10):899–912. doi:10.1056/NEJMoa2404143
2. Bergmark BA, Marston NA, Bramson CR, et al. Effect of Vupanorsen on Non-High-Density Lipoprotein Cholesterol Levels in Statin-Treated Patients With Elevated Cholesterol: TRANSLATE-TIMI 70. *Circulation*. May 3 2022;145(18):1377–1386. doi:10.1161/CIRCULATIONAHA.122.059266
3. Bergmark BA, Marston NA, Prohaska TA, et al. Olezarsen for Hypertriglyceridemia in Patients at High Cardiovascular Risk. *N Engl J Med*. May 16 2024;390(19):1770–1780. doi:10.1056/NEJMoa2402309
4. Fukuhara K, Furihata K, Matsuoka N, et al. A multi-purpose Japanese phase I study in the global development of vupanorsen: Randomized, placebo-controlled, single-ascending dose study in adults. *Clin Transl Sci*. May 2023;16(5):886–897. doi:10.1111/cts.13498
5. Gaudet D, Pall D, Watts GF, et al. Plozasiran (ARO-APOC3) for Severe Hypertriglyceridemia: The SHASTA-2 Randomized Clinical Trial. *JAMA Cardiol*. Jul 1 2024;9(7):620–630. doi:10.1001/jamacardio.2024.0959
6. Luo Z, Huang Z, Sun F, et al. The clinical effects of inclisiran, a first-in-class LDL-C lowering siRNA therapy, on the LDL-C levels in Chinese patients with hypercholesterolemia. *J Clin Lipidol*. May–Jun 2023;17(3):392–400. doi:10.1016/j.jacl.2023.04.010
7. Nissen SE, Wolski K, Balog C, et al. Single Ascending Dose Study of a Short Interfering RNA Targeting Lipoprotein(a) Production in Individuals With Elevated Plasma Lipoprotein(a) Levels. *JAMA*. May 3 2022;327(17):1679–1687. doi:10.1001/jama.2022.5050
8. Raal F, Durst R, Bi R, et al. Efficacy, Safety, and Tolerability of Inclisiran in Patients With Homozygous Familial Hypercholesterolemia: Results From the ORION-5 Randomized Clinical Trial. *Circulation*. Jan 30 2024;149(5):354–362. doi:10.1161/CIRCULATIONAHA.122.063460
9. Rekic D, Azarov I, Knochel J, et al. AZD8233 antisense oligonucleotide targeting PCSK9 does not prolong QT interval. *Br J Clin Pharmacol*. Nov 2022;88(11):4839–4844. doi:10.1111/bcp.15425
10. Rosenson RS, Gaudet D, Hegele RA, et al. Zodasiran, an RNAi Therapeutic Targeting ANGPTL3, for Mixed Hyperlipidemia. *N Engl J Med*. Sep 12 2024;391(10):913–925. doi:10.1056/NEJMoa2404147
11. Tardif JC, Karwatowska-Prokopczuk E, Amour ES, et al. Apolipoprotein C-III reduction in subjects with moderate hypertriglyceridaemia and at high cardiovascular risk. *Eur Heart J*. Apr 6 2022;43(14):1401–1412. doi:10.1093/eurheartj/ehab820
12. Zimerman A, Wiviott SD, Park JG, et al. Reductions in remnant cholesterol and VLDL cholesterol through inhibition of ANGPTL3 protein synthesis: an analysis from the TRANSLATE-TIMI 70 trial. *Eur J Prev Cardiol*. Aug 9 2024;31(10):1216–1223. doi:10.1093/eurjpc/zwae090
13. Barć P, Antkiewicz M, Śliwa B, et al. Double VEGF/HGF Gene Therapy in Critical Limb Ischemia Complicated by Diabetes Mellitus. *J Cardiovasc Transl Res*. Jun 2021;14(3):409–415. doi:10.1007/s12265-020-10066-9
14. Gu Y, Cui S, Liu C, et al. pUDK-HGF Gene Therapy to Relieve CLI Rest Pain and Ulcer: A Phase II, Double-Blind, Randomized Placebo-Controlled Trial. *Hum Gene Ther*. Aug 2021;32(15-16):839–849. doi:10.1089/hum.2020.290

15. Gu Y, Cui S, Wang Q, et al. A Randomized, Double-Blind, Placebo-Controlled Phase II Study of Hepatocyte Growth Factor in the Treatment of Critical Limb Ischemia. *Mol Ther*. Dec 4 2019;27(12):2158–2165. doi:10.1016/j.ymthe.2019.10.017
16. Hammad TA, Rundback J, Bunte M, et al. Stromal Cell-Derived Factor-1 Plasmid Treatment for Patients With Peripheral Artery Disease (STOP-PAD) Trial: Six-Month Results. *J Endovasc Ther*. Aug 2020;27(4):669–675. doi:10.1177/1526602820919951
17. Fontana M, Berk JL, Gillmore JD, et al. Vutrisiran in Patients with Transthyretin Amyloidosis with Cardiomyopathy. *N Engl J Med*. Jan 2 2025;392(1):33–44. doi:10.1056/NEJMoa2409134
18. Garcia-Pavia P, Grogan M, Kale P, et al. Impact of vutrisiran on exploratory cardiac parameters in hereditary transthyretin-mediated amyloidosis with polyneuropathy. *Eur J Heart Fail*. Feb 2024;26(2):397–410. doi:10.1002/ejhf.3138
19. Judge DP, Kristen AV, Grogan M, et al. Phase 3 Multicenter Study of Revusiran in Patients with Hereditary Transthyretin-Mediated (hATTR) Amyloidosis with Cardiomyopathy (ENDEAVOUR). *Cardiovasc Drugs Ther*. Jun 2020;34(3):357–370. doi:10.1007/s10557-019-06919-4
20. Maurer MS, Kale P, Fontana M, et al. Patisiran Treatment in Patients with Transthyretin Cardiac Amyloidosis. *N Engl J Med*. Oct 26 2023;389(17):1553–1565. doi:10.1056/NEJMoa2300757
21. Lyon AR, Babalis D, Morley-Smith AC, et al. Investigation of the safety and feasibility of AAV1/SERCA2a gene transfer in patients with chronic heart failure supported with a left ventricular assist device - the SERCA-LVAD TRIAL. *Gene Ther*. Dec 2020;27(12):579–590. doi:10.1038/s41434-020-0171-7
22. Taubel J, Hauke W, Rump S, et al. Novel antisense therapy targeting microRNA-132 in patients with heart failure: results of a first-in-human Phase 1b randomized, double-blind, placebo-controlled study. *Eur Heart J*. Jan 7 2021;42(2):178–188. doi:10.1093/eurheartj/ehaa898
23. Bakris GL, Saxena M, Gupta A, et al. RNA Interference With Zilebesiran for Mild to Moderate Hypertension: The KARDIA-1 Randomized Clinical Trial. *JAMA*. Mar 5 2024;331(9):740–749. doi:10.1001/jama.2024.0728
24. Desai AS, Webb DJ, Taubel J, et al. Zilebesiran, an RNA Interference Therapeutic Agent for Hypertension. *N Engl J Med*. Jul 20 2023;389(3):228–238. doi:10.1056/NEJMoa2208391
25. Gaudet D, Karwatowska-Prokopczuk E, Baum SJ, et al. Vupanorsen, an N-acetyl galactosamine-conjugated antisense drug to ANGPTL3 mRNA, lowers triglycerides and atherogenic lipoproteins in patients with diabetes, hepatic steatosis, and hypertriglyceridaemia. *Eur Heart J*. Oct 21 2020;41(40):3936–3945. doi:10.1093/eurheartj/ehaa689
26. Gouni-Berthold I, Alexander VJ, Yang Q, et al. Efficacy and safety of volanesorsen in patients with multifactorial chylomicronaemia (COMPASS): a multicentre, double-blind, randomised, placebo-controlled, phase 3 trial. *Lancet Diabetes Endocrinol*. May 2021;9(5):264–275. doi:10.1016/S2213-8587(21)00046-2
27. Leikas AJ, Hassinen I, Kivelä A, et al. Intramyocardial adenoviral vascular endothelial growth factor-D(ΔNΔC) gene therapy does not induce ventricular arrhythmias. *J Gene Med*. Aug 2022;24(8):e3437. doi:10.1002/jgm.3437
28. Leiter LA, Teoh H, Kallend D, et al. Inclisiran Lowers LDL-C and PCSK9 Irrespective of Diabetes Status: The ORION-1 Randomized Clinical Trial. *Diabetes Care*. Jan 2019;42(1):173–176. doi:10.2337/dc18-1491.

29. Ray KK, Landmesser U, Leiter LA, et al. Inclisiran in Patients at High Cardiovascular Risk with Elevated LDL Cholesterol. *N Engl J Med*. Apr 13 2017;376(15):1430–1440. doi:10.1056/NEJMoa1615758
